# RETRACTION: The Effect of the Cortical Bone Trajectory Screw Fixation and Traditional Pedicle Screw Fixation on Surgical Site Wound Infection in Posterior Lumbar Fusion Wound: A Meta‐Analysis

**DOI:** 10.1111/iwj.70203

**Published:** 2025-02-02

## Abstract

**RETRACTION**: MaoH. , WangZ. , and LiQ. , “The Effect of the Cortical Bone Trajectory Screw Fixation and Traditional Pedicle Screw Fixation on Surgical Site Wound Infection in Posterior Lumbar Fusion Wound: A Meta‐Analysis,” International Wound Journal 20, no. 8 (2023): 3241–3248, 10.1111/iwj.14203.37264722 PMC10502259

The above article, published online on 01 June 2023, in Wiley Online Library (http://onlinelibrary.wiley.com/), has been retracted by agreement between the journal Editor in Chief, Professor Keith Harding; and John Wiley & Sons Ltd. Following an investigation by the publisher, all parties have concluded that this article was accepted solely on the basis of a compromised peer review process. The editors have therefore decided to retract the article. The authors did not respond to our notice regarding the retraction.

**RETRACTION**: H. Mao , Z. Wang , and Q. Li , “The Effect of the Cortical Bone Trajectory Screw Fixation and Traditional Pedicle Screw Fixation on Surgical Site Wound Infection in Posterior Lumbar Fusion Wound: A Meta‐Analysis,” International Wound Journal 20, no. 8 (2023): 3241–3248, 10.1111/iwj.14203.37264722 PMC10502259

The above article, published online on 01 June 2023, in Wiley Online Library (http://onlinelibrary.wiley.com/), has been retracted by agreement between the journal Editor in Chief, Professor Keith Harding; and John Wiley & Sons Ltd. Following an investigation by the publisher, all parties have concluded that this article was accepted solely on the basis of a compromised peer review process. The editors have therefore decided to retract the article. The authors did not respond to our notice regarding the retraction.

